# From trauma exposure to depression or resilience: cognitive-emotional vulnerabilities and protective roles of rumination, emotion dysregulation, and self-compassion

**DOI:** 10.3389/fpsyt.2026.1919604

**Published:** 2026-09-21

**Authors:** Lin Ma

**Affiliations:** Student Affairs Department Mental Health Education Center, Harbin Normal University, Harbin, Heilongjiang, China

**Keywords:** depression, emotion dysregulation, resilience, rumination, self-compassion, trauma

## Abstract

Exposure to traumatic experiences is a well-documented risk factor for the development of depressive disorders; however, considerable variability exists in psychological outcomes following trauma exposure, with many individuals demonstrating resilience despite significant adversity. Understanding the mechanisms underlying these divergent trajectories is essential for improving prevention and intervention strategies. This narrative review integrates current empirical evidence on rumination, emotion dysregulation, and self-compassion to propose a cognitive-emotional framework explaining how maladaptive and protective processes may contribute to post-traumatic depression and resilience. The literature examined indicates that maladaptive rumination and difficulties in emotion regulation are consistently associated with elevated depressive symptoms following trauma and may contribute to the persistence of psychological distress through repetitive negative thinking, impaired emotional processing, and reduced adaptive coping. Conversely, self-compassion appears to be associated with lower levels of rumination, improved emotional functioning, and greater resilience. However, the extent to which self-compassion exerts unique effects beyond related processes such as mindfulness, acceptance, and broader emotion regulation capacities remains insufficiently understood. Overall, the available evidence supports a transdiagnostic perspective in which cognitive, emotional, and self-relational processes interact to influence post-traumatic adaptation. Nevertheless, the predominance of cross-sectional designs, reliance on self-report measures, and heterogeneity across trauma populations limit causal interpretations and the definitive identification of underlying mechanisms. Future longitudinal, experimental, and mechanistically informed studies are required to clarify the temporal relationships among these constructs and to determine how targeted interventions may strengthen protective processes while reducing vulnerability to depression following trauma.

## 1 Introduction

The exposure to events that may be deemed traumatic is exceedingly widespread on a global scale and constitutes a significant concern for public health. Epidemiological investigations reveal that a considerable proportion of individuals encounter at least one traumatic event throughout their lifetime, which may include interpersonal violence, childhood maltreatment, combat exposure, natural disasters, serious accidents, or life-threatening illnesses (1, 2). While exposure to trauma markedly elevates the likelihood of experiencing adverse psychological outcomes, individuals display notable variability in their responses to such traumatic experiences (3). In contrast to some trauma survivors who develop enduring psychopathology, others exhibit adaptive functioning and sustain psychological well-being in the face of considerable adversity (4). Elucidating the factors that facilitate these divergent trajectories continues to represent a primary challenge within the domain of contemporary trauma research. Among the myriad mental health repercussions linked to trauma, depression stands out as one of the most prevalent and debilitating consequences (5). Extensive research has consistently indicated that exposure to traumatic incidents correlates with a heightened susceptibility to major depressive disorder, recurrent episodes of depression, and enduring depressive symptoms throughout the human lifespan (6, 7). The occurrence of depression subsequent to trauma is linked to considerable functional impairment, increased utilization of healthcare resources, diminished quality of life, and an amplified risk of suicidal behaviors (8). Crucially, it is noteworthy that not all individuals exposed to trauma develop depressive disorders, indicating that additional cognitive, emotional, and interpersonal mechanisms play a significant role in post-traumatic adaptation (3, 9). The framework of resilience has emerged as a valuable paradigm for comprehending adaptive responses to adversity. While definitions of resilience differ across various studies, it is predominantly understood as the ability to sustain or recover psychological functioning when confronted with substantial stress or trauma (10). Modern interpretations underscore that resilience should not be regarded as a static personality trait; rather, it is a fluid and multidimensional process that encompasses interactions among biological, psychological, social, and environmental determinants (11). Accordingly, identifying modifiable cognitive and emotional processes that contribute to resilient adaptation has become an important objective in trauma research. Longitudinal studies have consistently revealed that resilience represents a prevalent trajectory following potentially traumatic experiences, with numerous individuals demonstrating stable psychological functioning despite enduring considerable adversity (4, 12). However, the underlying mechanisms that promote resilient adaptation are not yet fully elucidated. An expanding corpus of evidence indicates that cognitive-emotional processes are instrumental in influencing whether trauma survivors advance toward psychopathology or exhibit resilience (13). One such process is rumination, which is characterized as repetitive and passive contemplation of adverse experiences, emotions, and their repercussions (14). Rumination associated with trauma has been correlated with heightened depressive symptoms, extended emotional distress, and hindered recovery in the aftermath of traumatic events (15). Ongoing rumination may contribute to the maintenance of negative affect, reinforce maladaptive cognitive evaluations, and interfere with adaptive emotional processing, thereby potentially increasing vulnerability to depression (14, 15). Another salient mechanism is emotion dysregulation, which pertains to challenges in comprehending, modulating, and adapting responsively to emotional experiences (16). The exposure to traumatic events has been consistently associated with disruptions in emotional regulation abilities, which encompass increased emotional reactivity, experiential avoidance, and inadequacies in effective coping strategies (17). Emotion dysregulation has progressively been framed as a transdiagnostic phenomenon that underpins a wide array of psychological disorders, including depression and trauma-related psychopathology (16, 17). Challenges in the regulation of emotional responses may impede the rehabilitation process following trauma and contribute to the enduring nature of depressive symptoms over time. In contrast, self-compassion has emerged as a potentially important protective construct associated with adaptive adjustment following trauma exposure. Self-compassion entails treating oneself with benevolence during periods of suffering, acknowledging that adversities are an integral component of the shared human condition, and sustaining a balanced awareness of distressing thoughts and emotions (18). However, the mechanisms through which self-compassion exerts its effects remain incompletely understood, particularly given its conceptual overlap with mindfulness, acceptance-based processes, and broader emotion regulation capacities.

Accumulating empirical evidence indicates that self-compassion is correlated with diminished levels of depressive symptoms, decreased rumination, improved emotion regulation, and heightened resilience across a variety of populations (18, 19). Within populations of trauma survivors, self-compassion may foster adaptive coping mechanisms by mitigating self-criticism and encouraging emotional acceptance, thus serving as a buffer against detrimental psychological consequences (20). Notwithstanding the extensive research investigating rumination, emotion dysregulation, and self-compassion as discrete constructs, prior reviews have predominantly concentrated on these elements in isolation. Furthermore, limited attention has been given to how these processes may interact dynamically over time or whether self-compassion represents a distinct protective pathway versus an overlapping regulatory capacity shared with related constructs such as mindfulness. As a result, relatively scant attention has been directed towards elucidating the interactions between maladaptive cognitive-emotional processes and protective self-relational capacities that influence post-traumatic outcomes. An integrative theoretical framework may afford a more holistic comprehension of the differential pathways through which some trauma survivors experience depression while others exhibit resilience. This approach holds particular significance in light of the growing acknowledgment that psychological adaptation following traumatic experiences is shaped by intricate interactions among multiple risk and protective factors, rather than being attributable to singular mechanisms alone (11, 13). Consequently, the current narrative review endeavors to amalgamate the extant literature regarding the functions of rumination, emotional dysregulation, and self-compassion in the context of post-traumatic depression and resilience. In particular, this review aims to (1) collate empirical findings that associate these constructs with post-traumatic outcomes, (2) investigate potential mechanisms by which they affect vulnerability and resilience, and (3) propose a theoretically informed and empirically testable conceptual framework to enhance forthcoming research and inform targeted clinical interventions for individuals who have experienced trauma.

## 2 Methods

### 2.1 Literature search strategy

A narrative review methodology was utilized to synthesize and critically appraise the existing literature pertaining to the roles of rumination, emotional dysregulation, and self-compassion in the context of post-traumatic depression and resilience. This review was conducted as a narrative synthesis rather than a systematic review or meta-analysis; therefore, the primary objective was to critically integrate conceptual models, empirical findings, and mechanistic evidence rather than to quantitatively pool effect estimates. A structured literature search was performed across four prominent electronic databases: PubMed, Scopus, Web of Science, and PsycINFO. The search encompassed studies published from January 2000 through March 2026, thereby reflecting the increasing scholarly attention toward cognitive-emotional processes and compassion-related constructs within trauma research. The searches were restricted to articles published in the English language. The search strategy integrated terminology associated with trauma, depression, resilience, rumination, emotional regulation, and self-compassion using Boolean operators. The search strategy was intentionally broad to capture studies examining individual mechanisms and their interrelationships rather than requiring all constructs to be simultaneously investigated within a single study. The primary search terms included:

(“trauma” OR “psychological trauma” OR “adverse childhood experiences” OR “post-traumatic stress” OR “trauma survivors”) AND (“depression” OR “depressive symptoms” OR “major depressive disorder” OR “internalizing symptoms”) AND (“rumination” OR “repetitive negative thinking” OR “emotion dysregulation” OR “emotion regulation difficulties” OR “self-compassion” OR “compassion”).

Additional relevant studies were identified through manual screening of reference lists from retrieved articles and previous review papers.

### 2.2 Eligibility criteria

#### 2.2.1 Inclusion criteria

Studies were considered eligible if they met the following criteria:

- Included populations exposed to trauma or individuals who had experienced traumatic events;
- Investigated at least one of the primary constructs of interest, including rumination, emotional dysregulation, or self-compassion;
- Examined associations with depressive symptoms, major depressive disorder, resilience, or post-traumatic adjustment;
- Investigated psychological, cognitive, emotional, or neurobiological mechanisms that may explain relationships between trauma exposure and subsequent psychological outcomes;
- Utilized quantitative, qualitative, longitudinal, cross-sectional, experimental, or intervention-based methodologies;
- Were published in peer-reviewed academic journals;
- Were available in the English language.

#### 2.2.2 Exclusion criteria

Studies were excluded if they:

- Did not involve populations exposed to trauma;
- Focused exclusively on severe neurological disorders or psychotic disorders unrelated to trauma;
- Consisted of conference abstracts, dissertations, editorials, commentaries, or other non-peer-reviewed publications;
- Were unavailable in the English language;
- Failed to address any of the primary constructs of interest (rumination, emotional dysregulation, self-compassion, depression, or resilience).

### 2.3 Study selection and synthesis approach

Following database searches, titles and abstracts were screened for conceptual relevance to the objectives of this narrative review. Full-text articles considered potentially informative were subsequently evaluated to determine their contribution to understanding the relationships among trauma exposure, cognitive-emotional processes, and psychological outcomes. Because this review aimed to provide a critical and integrative synthesis of heterogeneous evidence, studies were not selected according to quantitative effect-size criteria, and a formal meta-analytic approach was not undertaken. The included studies were synthesized narratively and organized into three primary thematic domains:

1. rumination as a cognitive vulnerability factor,
2. emotion dysregulation as a transdiagnostic mechanism, and
3. self-compassion as a protective factor fostering resilience.

Particular emphasis was placed on identifying convergent findings, inconsistencies across studies, population-specific differences, and potential mechanisms linking trauma exposure with divergent psychological trajectories. An integrative conceptual framework was subsequently developed to illustrate how maladaptive cognitive-emotional processes and protective self-relational capacities may interact dynamically to influence pathways toward depression or resilience following trauma exposure. Given the narrative nature of this review and the absence of quantitative synthesis, PRISMA-based study selection procedures and formal risk-of-bias assessments were not applied. Instead, emphasis was placed on conceptual integration and critical interpretation of the available evidence.

## 3 Trauma and depression: conceptual and empirical background

### 3.1 Trauma as a risk factor for depression

Exposure to traumatic experiences not only heightens susceptibility to posttraumatic stress disorder (PTSD) but is increasingly acknowledged as a significant factor in the etiology of depressive psychopathology. Notably, the correlation between trauma and depression appears to exhibit a dose-dependent relationship, wherein repeated, chronic, or interpersonal forms of trauma present a considerably augmented risk as compared to isolated traumatic incidents. Specifically, adverse experiences in childhood including emotional maltreatment, neglect, and dysfunction within the household, have been demonstrated to have enduring impacts on emotional maturation and markedly increase the probability of depression in adulthood (21, 22). Empirical evidence derived from meta-analytic and prospective investigations further indicates that early traumatic experiences are linked to an earlier onset of depressive disorders, heightened symptom severity, elevated recurrence rates, and suboptimal treatment outcomes (5). The temporal dynamics of trauma exposure may significantly affect the manifestation of depressive outcomes. Exposure during critical developmental windows, particularly in childhood and adolescence, has the potential to disrupt normative neurodevelopmental trajectories and heighten vulnerability to subsequent stressors, a phenomenon frequently characterized as stress sensitization (23). Individuals subjected to early adversities may necessitate diminished environmental stress in later life to precipitate episodes of depression. Furthermore, trauma-associated depression commonly presents with distinctive clinical manifestations, encompassing heightened comorbidity, escalated suicidality, notable interpersonal challenges, and chronic illness trajectories (5, 24). Empirical investigations have further illuminated the cumulative impact of trauma. Research consistently indicates that exposure to multiple adverse experiences correlates with an incrementally greater risk of depressive symptoms, implying that cumulative adversity exerts additive or even synergistic effects on mental health outcomes (22, 25). Therefore, trauma exposure should not be regarded solely as a triggering event but rather as a critical developmental risk factor that informs susceptibility to depression across the lifespan. Emerging evidence further suggests that the psychological consequences of trauma may also be shaped by biological sex, sociocultural context, and socioeconomic conditions, highlighting that depression following trauma results from complex interactions between developmental, environmental, and individual vulnerability factors rather than trauma exposure alone.

### 3.2 Mechanisms linking trauma to depression

A multitude of interactive mechanisms have been posited to elucidate the manner in which traumatic experiences augment susceptibility to depressive disorders. A prominent theoretical framework underscores modifications in biological systems that respond to stress. The experience of trauma, particularly when it is chronic or occurs during early development, has been correlated with dysregulation of the hypothalamic-pituitary-adrenal (HPA) axis, leading to aberrant cortisol secretion patterns and compromised stress adaptation (26). The sustained activation of stress-response systems may contribute to an increased allostatic load, thus heightening the vulnerability to depressive illnesses. Furthermore, trauma has been associated with both structural and functional modifications in cerebral regions that are vital for mood regulation. Neuroimaging investigations have revealed significant alterations in the hippocampus, amygdala, and prefrontal cortex (PFC) among individuals exposed to trauma, which are regions fundamentally engaged in memory processing, emotional reactivity, and executive functioning (27). These neurobiological changes may undermine individuals’ capabilities to modulate affective responses and to process stressful experiences in an adaptive manner. Beyond changes confined to discrete brain regions, current neurocognitive frameworks increasingly highlight impairments within large-scale functional brain networks as a central pathway through which trauma may contribute to depression. Notably, the Default Mode Network (DMN), which underpins self-referential processing and autobiographical memory, has been repeatedly linked to repetitive negative thinking and dysfunctional rumination. Altered DMN connectivity in the context of trauma may foster enduring self-focused negative cognition and reduce the ability to disengage from distressing memories, thereby heightening susceptibility to depressive symptoms (28, 29). At the same time, the Salience Network (SN), centered on the anterior insula and anterior cingulate cortex, is essential for identifying emotionally relevant stimuli and for flexibly shifting between attention directed inward and attention directed outward. Reduced flexibility or suboptimal recruitment of the SN after trauma may hinder emotional awareness and adaptive control, thereby sustaining maladaptive rumination and extended negative affect (30). Impaired flexibility within this network may compromise emotion regulation and increase vulnerability to prolonged negative affect following trauma. Recent neuroimaging findings further suggest that depressive vulnerability is characterized not only by heightened negative self-referential processing involving DMN and SN regions but also by insufficient recruitment of systems supporting embodied self-processing, highlighting the interaction between internally focused cognition and emotional regulation in depressive psychopathology (31). Another pathway pertains to inflammatory mechanisms. Emerging research indicates that exposure to trauma is correlated with persistent low-grade inflammation, which is characterized by increased levels of circulating inflammatory biomarkers such as C-reactive protein, interleukin-6, and tumor necrosis factor-alpha (32). These inflammatory responses may exert an influence on neurotransmitter systems, neural plasticity, and reward processing, thereby contributing to the manifestation of depressive symptomatology. Rather than operating independently, neuroendocrine, inflammatory, and large-scale neural network alterations are increasingly recognized as interacting systems that collectively shape cognitive vulnerability, emotional dysregulation, and persistent depressive symptoms following trauma (33). Interpersonal mechanisms are also gaining recognition in this context. Trauma, especially that which involves interpersonal victimization, can disrupt the security of attachment and compromise social functioning (34). Diminished interpersonal trust, increased sensitivity to rejection, and social withdrawal may subsequently limit access to supportive relationships, which represent some of the most significant protective factors against depression. Consequently, biological, neurodevelopmental, large-scale neural network, and interpersonal pathways are likely to interact dynamically to modulate vulnerability to depression following trauma.

### 3.3 Why are outcomes heterogeneous?

One of the most compelling findings in the field of trauma research is that individuals subjected to similar traumatic experiences frequently demonstrate significantly divergent psychological trajectories. Contemporary theoretical frameworks increasingly regard post-traumatic adaptation as a result of intricate interactions among pre-trauma vulnerabilities, the characteristics of the trauma itself, and post-trauma environmental influences, rather than merely a direct outcome of trauma exposure (13). Pre-existing individual differences represent a crucial source of variability. Genetic predispositions, temperamental characteristics, personality dimensions, and previous psychiatric histories have all been empirically demonstrated to affect post-traumatic adjustment (35). For instance, elevated levels of neuroticism and existing affective disorders may intensify emotional reactions to adversity, while traits such as dispositional optimism and psychological resilience may facilitate more adaptive recovery. Similarly, variations in cognitive appraisals appear to hold particular significance. Individuals who perceive traumatic incidents as indicative of personal failure, irreversible loss, or pervasive threat may be at an increased risk of developing chronic distress (36). The characteristics inherent to the traumatic event itself significantly contribute to the observed heterogeneity. Importantly, the effects of trauma on later psychological outcomes are unlikely to be consistent across individuals and may be influenced by multiple contextual variables. The type of trauma and the timing of development seem especially important, because early-life and interpersonal traumas may interfere with emotional development and self-related processes more strongly than isolated traumatic events that occur later. Moreover, cultural context may shape how distress is expressed, the coping strategies that are used, and the extent to which social resources are available after trauma. Gender-related factors and socioeconomic conditions may also further affect vulnerability and resilience by determining patterns of exposure, capacities for emotion regulation, and access to supportive environments. Consequently, explaining post-traumatic depression and resilience requires attention not only to individual psychological mechanisms but also to wider developmental and sociocultural contexts. Interpersonal and intentional traumas, such as sexual assault or childhood maltreatment, typically present a higher risk for negative psychological outcomes when compared to accidental or natural disasters (13). The severity of the trauma, its chronic nature, the perceived threat to life, and cumulative exposure to traumatic experiences further shape psychological trajectories. Importantly, the psychological consequences of trauma are also influenced by contextual and individual-level moderators, including sociocultural background, gender-related factors, and socioeconomic adversity, which may shape access to protective resources, coping strategies, and long-term adaptation following trauma (13). Ultimately, the conditions present in the post-trauma environment may play a pivotal role in determining the recovery process. Social support consistently emerges as one of the most robust predictors of resilience in the aftermath of trauma (37). In contrast, persistent stressors, social isolation, and adverse socioeconomic conditions may hinder recovery and elevate the risk of developing depression. These findings emphasize that resilience and psychopathology ought to be conceptualized as dynamic processes that stem from ongoing interactions between individuals and their environments, rather than as static outcomes. Beyond the features of trauma exposure itself, accumulating evidence indicates that individual differences in cognitive and emotional processing may be crucial determinants of post-traumatic pathways. In particular, repetitive negative rumination, impairments in emotion regulation, and diminished ability for adaptive self-referential processing may help explain the persistence of depressive symptoms in people exposed to trauma (14). By contrast, psychological processes linked to flexible emotional awareness, self-directed compassion, and adaptive coping may promote resilience (16). However, these processes are unlikely to function in isolation and may instead represent the interplay of multiple mechanisms, including cognitive appraisal, attachment-related development, interoceptive awareness, and the activity of large-scale neural networks (38, 39). Accordingly, explaining why some individuals recover while others develop chronic depression calls for a multidimensional framework that integrates developmental, psychological, and neurobiological factors.

## 4 Rumination as a cognitive vulnerability factor

### 4.1 Definition and dimensions of rumination

Rumination is typically understood as a cyclical and persistent cognitive pattern concentrating on adverse emotions, psychological distress, as well as their underlying causes and ramifications, rather than engaging in proactive problem-solving strategies. While it was initially examined within the framework of depressive disorders, rumination has progressively been acknowledged as a transdiagnostic cognitive phenomenon associated with various forms of psychopathology, encompassing disorders related to trauma. Modern interpretations highlight that rumination should not be regarded as a singular construct; instead, it comprises qualitatively distinct dimensions which may exert differential effects on post-traumatic adjustment. Nolen-Hoeksema et al. (14) suggested that maladaptive rumination is characterized by passive, repetitive, and abstract cognitive processes that sustain negative affectivity. Subsequent investigations in trauma have revealed that the content, temporal attributes, and functional characteristics of rumination may hold greater significance than mere frequency in forecasting psychopathological outcomes (**Table 1**). In particular, Michael et al. identified that attributes such as compulsive involvement in rumination, repetitive inquiries framed as “why” and “what if,” unconstructive cognitive patterns, and exacerbated negative emotional states preceding and succeeding rumination were significantly correlated with the severity of post-traumatic stress disorder both concurrently and prospectively (15). Contemporary theoretical frameworks further differentiate among various forms of trauma-related rumination. A notably impactful distinction is that which separates intrusive rumination from deliberate rumination. Intrusive rumination is characterized by involuntary, repetitive, and unwarranted thoughts pertaining to the traumatic incident, whereas deliberate rumination encompasses intentional and reflective cognitive processing aimed at comprehending, integrating, and deriving meaning from the experience (40). Furthermore, Stockton et al. elucidated that brooding, which is marked by passive comparisons between one’s present circumstances and unachieved standards, is distinct from reflective pondering, which may promote adaptive meaning-making under certain circumstances (41). These results suggest that rumination ought to be understood as a complex cognitive phenomenon, the effect of which on psychological outcomes is contingent upon both its modality and purpose. In contrast to repetitive, abstract, and involuntary forms of rumination, which seem to be maladaptive, more reflective and intentional modes of cognitive processing may facilitate adaptation and personal development in the aftermath of trauma. The differential effects of rumination may therefore depend not only on its content and frequency but also on an individual’s capacity for cognitive flexibility and emotional regulation during trauma processing.

**Table 1:** Key studies examining rumination in trauma survivors.

Population/Trauma type	Design	Sample size	Findings	Study
Motor vehicle accident survivors	Prospective longitudinal	N = 967	Rumination predicted chronic and delayed PTSD.	(42)
Ambulance service workers	Cross-sectional	N = 56	Rumination was associated with greater PTSD severity.	(49)
Road traffic accident survivors	Prospective longitudinal	N = 27 and N = 176	Early rumination predicted chronic PTSD.	(43)
Assault survivors	Cross-sectional and prospective longitudinal	N = 81; N = 73	Maladaptive rumination predicted PTSD and triggered intrusions.	(15)
Road traffic accident survivors	Cross-sectional and prospective longitudinal	N = 101; N = 147	Rumination predicted both PTSD and depression.	(45)
Assault survivors	Prospective longitudinal	N = 203	Rumination mediated the development of PTSD and depression.	(46)
Individuals reporting traumatic/stressful events	Cross-sectional	US: N = 224; Japan: N = 431	Deliberate rumination was associated with posttraumatic growth.	(40)
Healthy adults exposed to analogue trauma	Experimental	N = 101	Rumination maintained negative mood and intrusions.	(50)
PTSD and depressed patients	Cross-sectional clinical study	N = 65	Trauma-exposed patients showed greater rumination.	(47)
Individuals with past traumatic experiences	Cross-sectional	Not reported	Deliberate rumination was linked to posttraumatic growth.	(41)
Adolescents and adults exposed to stress	Longitudinal	Adolescents: N = 1,065; Adults: N = 1,132	Rumination mediated stress-related depression and anxiety.	(44)
Trauma-exposed psychiatric patients	Cross-sectional	N = 45	Specific rumination styles strengthened PTSD-depression associations.	(48)

PTSD, posttraumatic stress disorder; US, United States.

### 4.2 Trauma-induced rumination

Traumatic experiences often elicit substantial cognitive activity as individuals endeavor to comprehend, articulate, and assimilate profoundly distressing occurrences into their pre-existing belief frameworks. Consequently, rumination may manifest as an immediate and ostensibly adaptive mechanism to process trauma-related information. Nonetheless, sustained engagement in maladaptive ruminative thought processes can obstruct recovery and exacerbate prolonged psychological distress. Empirical research indicates that individuals who have experienced trauma typically engage in both intrusive and intentional forms of rumination. Taku et al. (40) found that intrusive rumination occurring in the aftermath of trauma was positively correlated with posttraumatic growth (PTG), potentially indicating preliminary cognitive engagement with the traumatic event. However, deliberate rumination that transpires later in the recovery trajectory emerged as the most significant predictor of current PTG in both American and Japanese cohorts, underscoring the critical role of intentional cognitive processing in facilitating adaptive recovery. In a similar vein, Stockton et al. identified a positive correlation between deliberate rumination and reflective processing with PTG, while intrusive re-experiencing and brooding exhibited no significant association with growth outcomes (41). Notably, rumination induced by trauma does not uniformly yield beneficial effects. Longitudinal studies involving survivors of road traffic accidents revealed that ongoing rumination in the weeks and months subsequent to the traumatic event emerged as one of the most potent predictors of chronic PTSD symptomatology (42, 43). Specifically, rumination measured four weeks’ post-trauma was found to predict later symptom severity more effectively than numerous initial trauma responses (43). Additionally, empirical evidence indicates that exposure to stressful and traumatic events may facilitate the emergence of a ruminative cognitive style. In two extensive longitudinal studies, Michl et al. demonstrated that encounters with stressful life events prospectively forecasted increases in rumination over time, and that rumination subsequently mediated the connection between stress exposure and internalizing symptoms (44). The results of this investigation imply that trauma may not solely elicit temporary ruminative responses but also influence the formation of persistent maladaptive cognitive processing patterns. In summary, a growing body of evidence suggests that rumination induced by trauma constitutes a dynamic phenomenon wherein initial intrusive thoughts can develop into either adaptive intentional processing or maladaptive repetitive brooding, thereby ultimately affecting post-traumatic consequences. Thus, trauma-related rumination should be conceptualized as a dynamic process rather than a uniformly maladaptive response, with its psychological consequences shaped by timing, cognitive style, and emotional regulatory capacity.

### 4.3 Rumination and post-traumatic depression

A considerable corpus of empirical research delineates rumination as a significant cognitive vulnerability factor contributing to the onset of depression subsequent to trauma exposure. Within various trauma-affected populations, individuals who demonstrate frequent and maladaptive rumination consistently report elevated levels of depressive symptomatology and exhibit suboptimal psychological adjustment over time. Longitudinal evidence has substantiated that rumination serves as a predictor of subsequent depressive symptoms, even when controlling for initial symptom severity. In a longitudinal investigation involving survivors of road traffic accidents, Ehring et al. established that rumination significantly anticipated both PTSD and depression six months’ post-trauma, independent of initial symptom levels (45). Similarly, Kleim and Ehlers identified that diminished specificity in autobiographical memory shortly following an assault was predictive of major depression at the six-month mark, with rumination partially mediating this association (46). Clinical investigations further elucidate that rumination associated with trauma may be particularly pronounced among individuals who exhibit comorbid trauma-related psychopathology and depressive disorders. Birrer and Michael noted that patients with depression who have been exposed to trauma, as well as those diagnosed with PTSD, exhibited significantly higher levels of rumination compared to non-traumatized individuals suffering from depression (47). Furthermore, rumination infrequently mitigated psychological distress; rather, participants consistently reported exacerbated feelings subsequent to episodes of rumination (47). The association between PTSD and depressive symptoms may also be intensified by particular modalities of rumination. Roley et al. illustrated that both repetitive and anticipatory rumination served to moderate the relationship between PTSD and depressive symptoms, thereby implying that these cognitive patterns may contribute to the elevated rates of comorbidity observed between these two disorders (48). Collectively, these findings bolster the proposition that rumination appears to function not merely as a correlate of post-traumatic depression but also as a potential maintaining and exacerbating factor associated with persistent depressive symptoms following trauma.

### 4.4 Potential mechanisms

Numerous theoretical frameworks have been advanced to elucidate the mechanisms by which rumination exacerbates depression and engenders maladaptive post-traumatic outcomes. A significant pathway encompasses the reinforcement of negative cognitive biases. In accordance with the cognitive models articulated by Ehlers and Clark (36), rumination sustains excessively adverse evaluations of traumatic events by incessantly directing attention toward themes of threat, loss, guilt, and irreversible change. Ehlers et al. demonstrated that negative interpretations of intrusive memories, in conjunction with rumination, considerably enhanced the predictive capacity for chronic PTSD beyond the mere objective characteristics of trauma, with rumination emerging as one of the most robust long-term indicators of symptom persistence (42). In a similar vein, Clohessy and Ehlers illustrated that negative evaluations of intrusive memories, alongside rumination, suppression, and dissociation, exhibited a strong correlation with the severity of PTSD among ambulance personnel (49). A secondary mechanism pertains to the sustained activation of stress response pathways. Empirical findings indicate that rumination extends both emotional and physiological responses to distressing events. In a study simulating trauma, Zetsche et al. discovered that subjects subjected to a rumination condition exhibited significantly diminished recovery from trauma-induced melancholic affect compared to those placed in memory integration or distraction conditions, thereby underscoring the causal influence of rumination in perpetuating adverse emotional states (50). Prolonged activation of cognitive and affective systems associated with stress may subsequently heighten susceptibility to depressive disorders. Ultimately, rumination may hinder adaptive emotional processing as well as the integration of memory. Individuals who have experienced trauma and who partake in repetitive abstract cognition may circumvent the direct processing of trauma-associated emotions, consequently obstructing the successful incorporation of the traumatic memory into autobiographical memory frameworks. Clohessy and Ehlers posited that rumination, along with suppression and dissociation, may obstruct emotional processing and contribute to the persistence of psychopathological conditions (49). Similarly, Michael et al. observed that rumination frequently elicited intrusive memories rather than alleviating them, suggesting that it may paradoxically exacerbate trauma-related distress (15). In alignment with this perspective, rumination was found to partially mediate the connection between diminished autobiographical memory specificity and subsequent depression in survivors of assault (46). Collectively, these findings indicate that rumination may contribute substantially to post-traumatic depression through multiple interacting mechanisms through various interrelated mechanisms, encompassing negative cognitive biases, prolonged stress activation, and compromised emotional processing. Recent neurocognitive models further indicate that the persistence of trauma-related rumination may be, at least in part, explained by changes in large-scale brain networks that underlie self-referential processing and emotional regulation. Heightened involvement of the DMN, which supports internally oriented cognition and autobiographical processing, has been linked to repetitive negative thinking and maladaptive self-focused attention (29). In contrast, diminished flexibility in the SN, which coordinates the detection of emotionally relevant information and the shift between internal and external states, may hinder disengagement from ruminative loops and reduce the capacity to regulate distress effectively (30). Together, these network-level pathways offer a neurobiological basis for how trauma-related rumination may sustain ongoing depressive symptoms.

## 5 Emotion dysregulation and post-traumatic adjustment

### 5.1 Conceptualizing emotion dysregulation

Emotion regulation denotes the complex array of processes through which individuals systematically monitor, assess, and alter the intensity, duration, and manifestation of their emotional experiences to effectively attain personal objectives and adapt adeptly to the demands of their environment. In contrast, emotion dysregulation is characterized by challenges in comprehending, accepting, modulating, and flexibly responding to one’s emotional experiences. Instead of being defined as a singular deficiency, emotion dysregulation is increasingly understood as a multifaceted construct that encompasses a variety of interrelated processes, such as inadequate emotional awareness, restricted emotional clarity, nonacceptance of emotional responses, compromised impulse control during periods of distress, difficulties in pursuing goal-directed activities in the context of emotional arousal, and limited access to adaptive emotion regulation strategies (51, 52) (**Table 2**). Empirical evidence substantiates this multidimensional conceptualization. In a sample of undergraduates exposed to trauma, Tull et al. discovered that the severity of posttraumatic stress symptoms was significantly correlated with various dimensions of emotion dysregulation, which included deficits in emotional acceptance, diminished emotional clarity, challenges in sustaining goal-directed behavior during periods of distress, difficulties with impulse control, and restricted access to effective strategies for emotion regulation (51). Notably, individuals displaying probable PTSD exhibited markedly greater overall difficulties in emotion regulation compared to trauma-exposed individuals manifesting subthreshold symptoms (51). In a similar vein, Ehring and Quack indicated that trauma survivors with more pronounced PTSD symptoms encountered heightened challenges across nearly all domains of emotion regulation (52). Among the diverse dimensions explored, deficits in emotional clarity were identified as particularly strong and consistent correlates of posttraumatic symptomatology (52). Collectively, these empirical findings indicate that emotion dysregulation ought not to be conceptualized solely as an increase in emotional intensity or negative affect but, instead, as a comprehensive disruption in emotional functioning that impacts the manner in which trauma survivors navigate distressing internal experiences. Bradley et al. further elucidated that emotion dysregulation persisted as a significant correlate of psychiatric symptoms even when controlling for childhood trauma exposure and negative affect, thereby reinforcing its status as a distinct psychological construct rather than merely a superficial manifestation of generalized emotional distress (53). Importantly, contemporary models emphasize that emotion dysregulation reflects not only difficulties in controlling emotional responses but also impairments in emotional awareness, interpretation, and flexible adaptation to internal states. This broader conceptualization is particularly relevant in trauma-related psychopathology, where altered perception of emotional signals may influence subsequent cognitive processes such as rumination and maladaptive self-focused attention.

**Table 2:** Representative studies on emotion dysregulation following trauma.

Population/Trauma type	Design	Sample size	Findings	Study
Trauma-exposed undergraduates	Cross-sectional	N = 108	Emotion dysregulation was associated with PTS severity.	(51)
Trauma survivors	Cross-sectional	N = 616	PTSD severity was linked to emotion regulation difficulties.	(52)
SUD inpatients with/without PTSD	Cross-sectional	N = 206	Emotion dysregulation mediated PTSD–impulsivity associations.	(58)
Women with childhood abuse histories	Cross-sectional	N = 752	Emotion dysregulation mediated revictimization risk.	(59)
Women with abuse histories	Cross-sectional	N = 912	Childhood abuse predicted emotion dysregulation and PTSD symptoms.	(54)
Women with childhood abuse histories	Cross-sectional	N = 164	Emotion dysregulation predicted functional impairment.	(60)
Highly traumatized adults	Cross-sectional	N = 154	Emotion dysregulation partially mediated dissociation.	(106)
Trauma-exposed community adults	Cross-sectional	N = 530	Emotion dysregulation predicted multiple psychiatric symptoms.	(53)
Maltreated and non-maltreated children	Longitudinal	N = 421	Better emotion regulation predicted lower psychopathology.	(55)
Maltreated adolescents	Cross-sectional and longitudinal	N = 167; N = 439	Emotion regulation deficits mediated psychopathology risk.	(57)
Highly traumatized adults with/without PTSD	Neuroimaging cross-sectional	N = 51	PTSD was associated with reduced cingulum integrity.	(62)

PTSD, posttraumatic stress disorder; PTS, posttraumatic stress; SUD, substance use disorder.

### 5.2 Trauma and emotional dysregulation

A burgeoning corpus of research suggests that traumatic incidents, especially those occurring during critical developmental phases, significantly disrupt typical emotional maturation and heighten susceptibility to enduring difficulties in emotion regulation. While nearly all varieties of trauma may pose challenges to regulatory abilities, chronic and interpersonal types of victimization seem to be particularly harmful. Ehring and Quack investigated the challenges of emotion regulation among a substantial cohort of trauma survivors and discovered that individuals subjected to early-onset chronic interpersonal trauma reported heightened difficulties in emotion regulation compared to those who experienced isolated events or traumas that manifested later in life (52). Although certain discrepancies between groups diminished upon adjusting for the severity of PTSD, deficits in emotional clarity remained notably pronounced among survivors of chronic interpersonal trauma (52). Research directed towards childhood maltreatment has yielded further empirical support for these associations. Burns et al. revealed that females possessing histories of sexual, physical, or emotional abuse manifested significantly heightened difficulties in emotion regulation compared to their non-abused counterparts (54). Importantly, emotional abuse was identified as the most robust predictor of emotion dysregulation, indicating that recurrent invalidation and emotional maltreatment may impose particularly detrimental effects on emotional development. Moreover, emotion dysregulation was found to partially mediate the association between childhood abuse and posttraumatic stress symptoms, underscoring its potential function as a mechanism that links trauma exposure to subsequent psychopathology (54). Longitudinal research further indicates that trauma-related disruptions in emotional regulation may confer lasting developmental ramifications. In a prospective investigation, Kim and Cicchetti established that neglect, physical abuse, sexual abuse, exposure to various maltreatment subtypes, and earlier onset of trauma were all correlated with diminished capacities for emotion regulation (55). Significantly, children exhibiting elevated levels of emotional regulation subsequently encountered enhanced peer acceptance and diminished internalizing psychopathology over an extended period, whereas inadequacies in emotional regulation forecasted maladaptive developmental trajectories (55). Theoretical frameworks concerning the development of trauma suggest that recurrent exposure to threatening or unpredictable caregiving contexts may disrupt the acquisition of adaptive emotional competencies, consequently heightening long-term susceptibility to affective disorders and maladaptive coping mechanisms (56). Such developmental disruptions may also affect the emergence of capacities considered important for adaptive emotional processing, including present-moment awareness, interoceptive sensitivity, and flexible self-related evaluation, which may partly explain why individuals exposed to similar traumatic experiences show divergent trajectories of recovery.

### 5.3 Emotion dysregulation as a transdiagnostic mechanism

Emotion dysregulation is increasingly regarded as a transdiagnostic phenomenon that plays a significant role in a variety of psychopathological conditions, rather than being confined to a specific diagnostic category. Individuals who have experienced trauma often exhibit intricate clinical presentations marked by the coexistence of PTSD, depression, substance abuse, dissociative symptoms, and behavioral dysregulation, which suggests that common deficiencies in emotion regulation may be a fundamental factor contributing to these varied manifestations. A considerable body of empirical research substantiates this viewpoint. Bradley et al. revealed that emotion dysregulation had a distinctive contribution to a diverse array of psychiatric symptoms, encompassing PTSD, depression, substance misuse, compromised functioning, and suicidal ideation, even when controlling for the effects of childhood trauma exposure and negative emotional states (53). These findings imply that emotion dysregulation may serve as a pivotal mechanism that underpins widespread psychiatric susceptibility. Further empirical support is derived from developmental research. In two distinct adolescent cohorts, Heleniak et al. ascertained that experiences of childhood maltreatment were predictive of elevated emotional reactivity, increased levels of rumination, and a propensity for impulsive reactions to distress (57). Furthermore, these deficits in emotion regulation served as mediators in the relationship between maltreatment and both internalizing and externalizing forms of psychopathology, thereby reinforcing the concept that disruptions in emotional regulation represent a transdiagnostic developmental pathway that connects traumatic experiences to a range of psychiatric outcomes (57). Additionally, emotion dysregulation has been associated with maladaptive behavioral responses following exposure to trauma. Weiss et al. demonstrated that emotion dysregulation completely mediated the relationship between PTSD and impulsive behaviors among individuals undergoing treatment for substance use disorders (58). Similarly, Messman-Moore et al. indicated that emotion dysregulation acted as a mediator in the relationships between childhood abuse and subsequent sexual revictimization, implying that challenges in managing emotional distress may heighten the likelihood of engaging in risky behaviors that further hinder psychological adjustment (59). Taken together, these findings suggest that emotion dysregulation may represent a central pathway through which trauma-related cognitive and affective processes interact. Rather than functioning as an isolated symptom domain, emotion dysregulation may amplify rumination, reduce cognitive flexibility, and limit access to adaptive coping responses, thereby increasing vulnerability to depression and other trauma-related disorders.

### 5.4 Associations with depression and resilience

Emotion dysregulation has been consistently recognized as a fundamental mechanism that connects trauma exposure to depressive outcomes. Individuals who have survived traumatic experiences and exhibit ongoing challenges in comprehending, accepting, and regulating their emotional responses are at an increased risk of developing depressive symptoms following adverse experiences. In an extensive community study, Bradley et al. illustrated that emotion dysregulation played a unique role in the severity of depressive symptoms, even after accounting for childhood trauma exposure and negative affect, thereby underscoring its incremental importance in post-traumatic psychopathology (53). In a similar vein, Heleniak et al. established that deficits in emotion regulation functioned as mediators in the relationship between childhood maltreatment and subsequent internalizing psychopathology, inclusive of depressive symptoms, across two distinct adolescent cohorts (57). Furthermore, empirical evidence indicates that emotion dysregulation may serve as a partial explanatory factor for the differential development of chronic psychopathology in some trauma survivors, while others exhibit resilient adaptation. Longitudinal research conducted by Kim and Cicchetti elucidated that elevated capacities for emotion regulation are predictive of enhanced peer acceptance and diminished internalizing symptoms over time, thereby suggesting that proficient emotion regulation may serve as a protective mechanism that fosters resilience in the aftermath of adverse experiences (55). In a similar vein, Cloitre et al. indicated that competencies in emotion regulation significantly contributed to functional adjustment in individuals who have endured childhood abuse, irrespective of the severity of PTSD symptoms (60). These empirical findings imply that adaptive emotional regulation competencies might facilitate recovery by improving social functioning, encouraging flexible coping strategies, and mitigating susceptibility to depressive responses. Contemporary theoretical frameworks of resilience posit that resilience transcends the mere absence of psychopathology, functioning instead as a dynamic process that entails successful emotional adaptation amidst adversities (3). Viewed through this lens, individual variability in emotion regulation capacity may constitute one of the most critical determinants influencing post-traumatic trajectories. However, the mechanisms through which some trauma-exposed individuals develop more adaptive regulatory capacities remain incompletely understood. Emerging evidence suggests that factors such as attachment security, interoceptive awareness, and the ability to adopt a nonjudgmental stance toward internal experiences may contribute to emotional adaptation, raising important questions regarding the specific mechanisms underlying resilience-enhancing interventions.

### 5.5 Possible neurobiological correlates

Neurobiological investigations suggest that dysregulation of emotions associated with trauma is linked to modifications in neural networks that govern emotional reactivity, cognitive regulation, and the management of stress. Within these networks, the amygdala, PFC, and the HPA axis have garnered significant scholarly attention. The amygdala is fundamentally instrumental in the identification of threats and the subsequent generation of emotional responses. The experience of trauma and the diagnosis of PTSD have been frequently correlated with an amplified reactivity of the amygdala, which may facilitate heightened emotional responses and enduring hypervigilance. Functional neuroimaging research has consistently revealed increased activation of the amygdala in individuals exposed to trauma who encounter challenges in regulating negative emotions (61). Conversely, the PFC, especially its medial and dorsolateral regions, plays a pivotal role in the top-down modulation of emotional responses. Diminished regulatory control from the PFC may hinder an individual’s capacity to attenuate negative affect subsequent to trauma exposure. Structural neuroimaging outcomes articulated by Fani et al. indicated a compromised integrity of the cingulum white matter tract among individuals diagnosed with PTSD when contrasted with trauma-exposed control subjects, thereby implying disrupted intercommunication between cerebral regions implicated in emotional processing and regulation (62). Modifications in the HPA axis have also been associated with emotional dysregulation linked to trauma. Prolonged exposure to traumatic experiences may result in dysregulated secretion of cortisol and hindered physiological recovery from stress, consequently heightening vulnerability to depressive disorders and enduring emotional turmoil. These neuroendocrine irregularities may interact with neural modifications within corticolimbic circuits to perpetuate maladaptive emotional responses in the aftermath of trauma (63). Beyond regional abnormalities, accumulating evidence suggests that trauma-related emotion dysregulation is characterized by disrupted communication among large-scale brain networks. The SN, comprising the anterior insula and anterior cingulate cortex, is central for identifying emotionally relevant internal and external signals and for coordinating adaptive responses. Malfunctioning within this network may compromise emotional awareness and flexible control after trauma (30). Moreover, changes in connectivity between the DMN and regulatory networks may foster enduring self-referential thought and make it harder to disengage from negative emotional conditions, thereby connecting emotion dysregulation with rumination and depressive vulnerability (29). From a transdiagnostic standpoint, altered interactions between the default mode network and the salience and regulatory systems may offer a neural basis through which trauma-related repetitive negative thinking becomes persistent and heightened in emotional impact. Recent neuroimaging findings further substantiate this network-based account by showing that depressive rumination entails both sustained involvement of self-referential systems and diminished engagement of neural systems that support embodied emotional regulation. Wu et al. (31) found that individuals with depression showed increased persistence of rumination-related activity in the default mode network, along with less recruitment of the insula and the SN. Crucially, people who were more capable of disengaging from rumination through greater recruitment of interoceptive processes reported fewer depressive symptoms and had a reduced risk of relapse. Taken together, these results indicate that maladaptive rumination may arise from an imbalance between excessive default mode network–mediated self-referential processing and insufficient activation of interoceptive and salience systems that underlie emotional awareness and adaptive regulation. In the setting of trauma-related psychopathology, such network-level dysconnectivity may constitute an important mechanism linking repetitive negative thinking, emotion dysregulation, and enduring vulnerability to depression. Excessive involvement of self-referential networks, together with diminished regulatory flexibility, may help sustain maladaptive interpretations of traumatic experiences, thereby increasing susceptibility to depressive symptoms. Nonetheless, it remains unclear whether these network-level changes are causal factors, downstream effects, or ongoing maintaining mechanisms in trauma-related psychopathology; this question requires longitudinal research (29, 30, 64). Consistent with this network-level perspective, the insula may serve as a critical interface between interoceptive processing and emotion regulation, suggesting that impaired awareness of bodily emotional cues may represent an additional pathway through which trauma disrupts adaptive emotional functioning (39).

## 6 Self-compassion as a protective factor

### 6.1 Definition and components of self-compassion

Self-compassion has emerged as a significant protective psychological resource in the framework of trauma exposure and subsequent recovery. According to Neff’s seminal conceptualization, self-compassion entails engaging in self-kindness and understanding during periods of suffering, rather than resorting to harsh self-criticism. The construct encompasses three interrelated dimensions: self-kindness in contrast to self-judgment, the recognition of common humanity as opposed to feelings of isolation, and mindfulness in comparison to over-identification with distressing experiences (65). Importantly, although mindfulness is included as one element within Neff’s framework for self-compassion, these constructs should not be treated as interchangeable. Mindfulness mainly denotes a style of attention and awareness marked by observation of the present moment, whereas self-compassion also entails an affective and motivational stance toward oneself during times of distress, encompassing warmth, care, and lessened self-criticism. Even so, there is considerable conceptual overlap, and existing evidence does not permit a full disentangling of the processes by which mindfulness-related awareness and compassionate self-relating support psychological adjustment (65, 66). These dimensions are posited to promote adaptive self-regulation by enabling individuals to acknowledge painful experiences without succumbing to overwhelming emotions or excessive avoidance (65, 67). Self-kindness pertains to the act of responding to personal failures or suffering with warmth, care, and understanding, rather than with criticism. The recognition of common humanity involves acknowledging that suffering and imperfection are universal facets of the human condition, thereby mitigating feelings of isolation. Mindfulness, the third dimension, involves sustaining a balanced awareness of painful thoughts and emotions without resorting to suppression, exaggeration, or fusion with them. The aforementioned components collectively may promote emotional equilibrium and psychological adaptability in the aftermath of traumatic events (65, 68). The significance of self-compassion in the context of trauma adaptation has garnered increasing empirical scrutiny. Research conducted by Thompson and Waltz revealed that heightened self-compassion correlates with diminished avoidance symptoms among individuals exposed to trauma, indicating that those who exhibit self-compassion may be less inclined to engage in experiential avoidance subsequent to trauma exposure (69). In a similar vein, Hiraoka et al. characterized self-compassion as a potentially modifiable construct, defined by attributes such as self-kindness, a sense of common humanity, and mindful awareness, which may significantly impact the emergence and persistence of trauma-related symptoms (70). In summary, these findings substantiate the conceptualization of self-compassion as a multidimensional construct with profound implications for post-traumatic adjustment (**Table 3**). However, an important conceptual challenge remains regarding the specificity of self-compassion as an active mechanism. Because self-compassion interventions frequently incorporate mindfulness, acceptance, and broader emotion regulation components, the extent to which observed benefits are uniquely attributable to compassionate self-relating versus overlapping regulatory processes remains unresolved.

**Table 3:** Empirical evidence linking self-compassion to post-traumatic outcomes.

Sample/Population	Study design	Main variables	Findings	Study
100 trauma-exposed university students	Cross-sectional	Self-compassion, PTSD symptoms	Higher self-compassion was associated with lower PTSD avoidance symptoms.	(69)
81 treatment-seeking youth with substance use problems and maltreatment histories	Cross-sectional	Childhood maltreatment, self-compassion, emotion dysregulation	Self-compassion mediated the association between childhood maltreatment and emotion dysregulation.	(80)
Community sample exposed to potentially traumatic events	Cross-sectional mediation study	Social support, self-compassion, PTSD, depression, anxiety	Self-compassion mediated associations between social support and PTSD, depression, and anxiety symptoms.	(73)
64 adolescents exposed to a wildfire disaster	Longitudinal (6-month follow-up)	Self-compassion, PTSD, depression, panic, suicidality	Self-compassion prospectively predicted lower trauma-related psychopathology.	(20)
115 Iraq and Afghanistan war veterans	Prospective longitudinal (12-month follow-up)	Self-compassion, PTSD symptoms, psychological health	Baseline self-compassion predicted lower PTSD symptom severity at follow-up.	(70)
453 trauma-exposed university students	Cross-sectional (SEM)	Self-compassion, PTSD symptoms, psychological health	Self-compassion was associated with better psychological health independent of PTSD severity.	(77)
Treatment-seeking women with PTSD and severe interpersonal trauma histories	Cross-sectional	Self-compassion, resilience, emotion dysregulation, PTSD	Self-compassion was positively associated with resilience and negatively associated with PTSD and emotion dysregulation.	(76)
48 patients with major depressive disorder	Experimental study	Self-compassion, depressed mood, emotion regulation	Self-compassion effectively reduced depressed mood and functioned as an adaptive emotion regulation strategy.	(74)

PTSD, posttraumatic stress disorder; SEM, structural equation modeling.

### 6.2 Self-compassion and trauma recovery

An increasing body of empirical evidence indicates that self-compassion serves a crucial function in promoting recovery subsequent to traumatic events. Survivors of trauma frequently contend with self-blame, shame, avoidance, and emotional distress, all of which may hinder constructive recovery mechanisms. Self-compassion may mitigate these maladaptive responses by reducing self-directed hostility, shame, and harsh self-evaluation, while promoting a more supportive stance toward one’s own suffering. These processes may indirectly facilitate adaptive coping and emotional processing. Preliminary findings from Thompson and Waltz established a negative correlation between self-compassion and avoidance symptoms in individuals exposed to trauma (69). Given that avoidance is regarded as a pivotal maintaining factor in post-traumatic psychopathology, a diminished reliance on avoidant coping strategies among individuals exhibiting high levels of self-compassion may facilitate the natural processing of emotions and recovery following trauma (69). Longitudinal studies further corroborate the protective function of self-compassion. In a sample of adolescents directly impacted by the Mount Carmel Forest Fire disaster, Zeller et al. illustrated that self-compassion was a prospective predictor of reduced levels of posttraumatic stress symptoms, depressive symptoms, panic symptoms, and suicidality across a six-month follow-up period, after accounting for dispositional mindfulness. These findings suggest that self-compassion may represent a potential protective factor in trauma recovery; however, whether self-compassion itself constitutes the active mechanism or reflects broader adaptive capacities such as mindfulness, emotional awareness, or psychological flexibility remains an important unresolved question (20). Further corroborative evidence is derived from investigations involving veterans who have experienced trauma. Hiraoka et al. demonstrated that initial levels of self-compassion were predictive of reduced PTSD symptom severity 12 months’ post-exposure, even when accounting for combat exposure and initial PTSD severity (70). Importantly, self-compassion should not be assumed to be a consistently available psychological resource across all trauma survivors. Its emergence and expression may be shaped by developmental experiences, attachment-related processes, and the maturation of emotion regulation abilities. People who experience chronic interpersonal adversity, especially during sensitive developmental periods, may face greater challenges in cultivating a compassionate way of relating to themselves because of disruptions in self-soothing, emotional awareness, and the flexible regulation of distress. Consequently, the higher levels of self-compassion found in some trauma survivors may indicate not only a protective factor but also retained adaptive regulatory capacities that support recovery after trauma (27, 71). This prospective correlation suggests that self-compassion may provide lasting protective benefits during the recovery process from trauma (70). Modern trauma frameworks increasingly regard self-compassion as an intrinsic resilience asset that may enhance recovery by mitigating feelings of shame, augmenting emotional acceptance, and facilitating adaptive meaning-making in the aftermath of adversity (19, 72).

### 6.3 Associations with depression

Self-compassion has been systematically correlated with diminished levels of depressive symptomatology in both populations exposed to trauma and clinical cohorts. Individuals exhibiting high levels of self-compassion tend to be less prone to react to distress through mechanisms such as self-criticism, rumination, or emotional avoidance, which are processes that have been robustly linked to the onset of depression. In a community-based sample comprising trauma-exposed individuals, Maheux and Price demonstrated that self-compassion was inversely correlated with symptoms associated with PTSD, generalized anxiety disorder, and depression (73). Additionally, self-compassion served as a mediating factor in the relationship between social support and depressive symptoms, indicating that social resources may exert advantageous effects in part by enhancing compassionate self-relating (73). Experimental findings further elucidate the potential of self-compassion as an antidepressant intervention. Diedrich et al. conducted a comparative analysis of self-compassion against cognitive reappraisal, acceptance, and a control condition among individuals diagnosed with major depressive disorder (74). The implementation of self-compassion yielded significantly greater reductions in depressive symptoms when contrasted with the control condition and demonstrated efficacy that was comparable to both cognitive reappraisal and acceptance methodologies. Notably, self-compassion was found to be particularly advantageous for individuals exhibiting elevated baseline levels of depressive symptoms (74). Theoretical frameworks suggest that self-compassion may serve as a protective mechanism against depression by disrupting maladaptive cognitive-emotional patterns typified by self-criticism, shame, and ruminative negative thought processes (19, 75). Considering the significant involvement of these processes in the context of post-traumatic depression, self-compassion may be regarded as an especially pertinent protective factor for individuals who have experienced trauma.

### 6.4 Self-compassion and resilience

Resilience in the aftermath of trauma pertains to the ability to sustain or restore psychological functionality despite the experience of adverse conditions. An increasing body of evidence suggests that self-compassion is a significant factor that facilitates resilience. Longitudinal research conducted by Zeller et al. revealed that self-compassion was a prospective predictor of reduced trauma-related psychopathology among adolescents exposed to disasters, thereby corroborating its role in promoting resilient adaptation (20). In a similar vein, Scoglio et al. identified that self-compassion exhibited a positive correlation with resilience and a negative correlation with both the severity of PTSD symptoms and emotional dysregulation in women seeking treatment who have a history of severe interpersonal trauma (76). Additional evidence is provided by Seligowski et al., who documented that the positive facets of self-compassion exhibited a robust correlation with enhanced overall psychological well-being among university students subjected to trauma, even when controlling for symptoms of PTSD and psychological inflexibility. These results imply that self-compassion plays a role in fostering positive psychological functioning that transcends the mere alleviation of psychopathological symptoms (77). It is critical to recognize that resilience should not be viewed merely as the absence of psychological distress, but rather as an intricate process of effective adaptation (9). Self-compassion may facilitate this adaptive process by promoting balanced emotional responses, nurturing hope, and bolstering effective coping mechanisms during challenging circumstances (72, 78). However, a key unresolved question is why capacities for self-compassion are more readily available to some trauma survivors than to others. Developmental factors, including attachment security, early caregiving experiences, the timing and chronicity of trauma exposure, and the preservation of emotional awareness capacities, may influence the degree to which individuals can engage in compassionate self-relating following adversity. Trauma survivors exposed to early interpersonal trauma may experience greater difficulty accessing self-compassion, as sustained exposure to threat may interfere with the development of self-soothing abilities, trust in internal experiences, and adaptive processing of emotional states. Consistent with this perspective, emerging evidence suggests that childhood trauma may be associated with alterations in mindfulness-related capacities, particularly acceptance and nonjudgmental awareness, which may influence vulnerability to later psychological symptoms (79). These findings raise the possibility that self-compassion-related processes may partly depend on overlapping capacities involving emotional awareness, acceptance, and nonreactive engagement with internal experiences. Thus, self-compassion should not be conceptualized as an equally available protective resource across trauma survivors; rather, its effectiveness may depend on developmental history, emotional awareness, and the capacity to safely engage with internal experiences.

### 6.5 Potential mechanisms of action

Several theoretical frameworks may elucidate the protective mechanisms afforded by self-compassion in the aftermath of trauma. A significant pathway pertains to the regulation of emotions. Vettese et al. established that self-compassion exhibited a negative correlation with difficulties in emotion regulation and served as a mediating factor in the relationship between the severity of childhood maltreatment and subsequent emotional dysregulation (80). Similarly, Scoglio et al. illustrated that emotional dysregulation acted as a mediator in the correlation between the severity of PTSD symptoms and self-compassion, thereby influencing the relationship between self-compassion and resilience (76). These findings imply that self-compassion may engender favorable outcomes by enhancing emotional regulation competencies. An additional mechanism may comprise diminished experiential avoidance. Thompson and Waltz discovered that elevated levels of self-compassion were distinctly correlated with reduced avoidance symptoms following exposure to traumatic events (69). The diminishment of avoidance may facilitate the emotional processing of traumatic experiences and mitigate the persistence of trauma-related distress (69). Social processes may also play a significant role. Maheux and Price elucidated that self-compassion served as a mediating variable in the association between social support and the manifestation of PTSD, anxiety, and depression in individuals exposed to trauma. These findings imply that external interpersonal resources may enhance internal compassionate self-relating, which subsequently fosters psychological adjustment (73). Moreover, interoceptive awareness may serve as an additional pathway that connects self-compassion to adaptive emotional functioning. Interoception—defined as the perception and interpretation of internal bodily cues—underpins emotional awareness and supports effective modulation of affective states. Developmental experiences, including attachment-related processes, may influence interoceptive functioning and the capacity to recognize and regulate emotional experiences (81). Moreover, emerging evidence suggests that interoceptive abilities are associated with emotion regulation processes, indicating that awareness of internal bodily states may provide an important foundation for adaptive emotional responding (82). After trauma, disrupted interoceptive processing may help explain problems in recognizing internal emotional states and in pursuing adaptive regulatory responses. In contrast, self-compassion practices may strengthen mindful awareness of internal experiences and promote a more accepting stance toward distressing emotional states. Nevertheless, it remains an open question whether these effects are specifically driven by self-compassion or instead reflect shared mechanisms with mindfulness-based approaches (39). Additional mechanisms identified in the scholarly literature comprise reductions in shame and self-criticism, augmentation of psychological flexibility, mitigation of rumination, and facilitation of constructive cognitive reappraisal processes (65, 72, 74). Collectively, these mechanisms may elucidate the rationale behind self-compassion fostering resilience while concurrently diminishing susceptibility to depression in the aftermath of trauma (19). From a neurocognitive standpoint, the mechanisms that support self-compassion are still not fully understood. Newer models indicate that compassionate self-relating may depend on interplay among systems involved in self-referential processing, detecting emotional salience, and maintaining interoceptive awareness (83, 84). For instance, greater engagement of regulatory networks and better integration of internal bodily cues could promote emotional awareness and limit routine involvement in maladaptive rumination (39, 84). Nevertheless, it is not yet clear whether these benefits are specifically driven by self-compassion or instead arise from partially overlapping mechanisms also present in mindfulness-based practices, acceptance-oriented approaches, and wider emotion regulation training (83, 85). Mechanistically guided future research is needed to separate these pathways.

## 7 An integrative model: pathways from trauma to depression and resilience

### 7.1 Interactions between rumination and emotion dysregulation

Although the phenomena of rumination and emotional dysregulation have traditionally been examined in isolation, emerging empirical evidence indicates that these processes are intricately interconnected in influencing post-traumatic outcomes. Rather than acting as discrete vulnerability factors, they may reinforce each other, engendering self-sustaining maladaptive cycles subsequent to trauma exposure. Rumination related to trauma may contribute to sustained emotional arousal by repeatedly engaging traumatic memories and negative self-referential cognitions and negative self-referential cognitions, thereby increasing the demands placed on regulatory systems (86). In turn, challenges in regulating intensified emotional states may foster additional engagement in repetitive negative thinking as individuals endeavor to comprehend, suppress, or exert control over distressing experiences (16). Adopting a process-oriented framework, rumination may be construed as a maladaptive cognitive strategy employed to navigate overwhelming affect, whereas emotional dysregulation signifies inadequacies in the effective modulation of that affect. Thus, persistent ruminative thought may deplete regulatory resources, diminish emotional clarity, and limit access to adaptive coping mechanisms (14). Conversely, individuals exhibiting inadequate emotional awareness or diminished regulatory capacity may exhibit a greater reliance on repetitive cognitive processing, as they are deficient in alternative strategies for addressing distress (87). The reciprocal association between rumination and emotion dysregulation may represent a self-reinforcing process that is associated with prolonged negative affect and increased vulnerability to depression following trauma exposure (88). In contrast, individuals who have experienced trauma and successfully disrupt this reciprocal cycle may demonstrate an increased likelihood of engaging in adaptive emotional processing and sustaining psychological well-being. Therefore, conceptualizing rumination and emotion dysregulation as interrelated processes rather than distinct mechanisms may yield a more nuanced understanding of post-traumatic heterogeneity and delineate multiple intervention targets aimed at preventing depressive trajectories. Importantly, the directionality and temporal sequence of this reciprocal relationship remain incompletely established. Longitudinal studies are required to determine whether rumination primarily precedes emotional dysregulation, whether regulatory deficits promote repetitive negative thinking, or whether these processes mutually intensify over time.

### 7.2 The moderating and buffering role of self-compassion

Self-compassion may represent a potentially important protective process that has the capacity to alter the effects of both rumination and emotional dysregulation on post-traumatic adaptation. Specifically, self-compassion may mitigate maladaptive cognitive-emotional cascades by cultivating a nonjudgmental and accepting orientation toward distress. Although these processes overlap with mindfulness-related capacities, self-compassion additionally involves a motivational component characterized by warmth and care toward oneself during suffering. According to models grounded in compassion, individuals who exhibit high levels of self-compassion are less inclined to become ensnared in self-critical or shame-based cognitive frameworks subsequent to adverse experiences (75). In the context of trauma, self-compassion may influence the strength of associations between maladaptive cognitive-emotional processes and depressive outcomes in the relationship between maladaptive cognitive-emotional processes and depressive outcomes. For instance, trauma survivors who demonstrate heightened rumination may encounter less severe depressive symptoms when they simultaneously possess elevated levels of self-compassion, as compassionate self-relating may attenuate identification with negative thoughts and diminish the emotional ramifications of recurrent thinking (89). Likewise, self-compassion may provide a buffer against emotional dysregulation by reducing secondary negative reactions to distress, including shame, self-criticism, and self-blame, thereby allowing individuals to respond to painful experiences with greater psychological flexibility (90). Another potential explanation is that self-compassion may function indirectly by enhancing psychological flexibility. However, these proposed pathways should not be interpreted as evidence that self-compassion operates through a single isolated mechanism. Rather, self-compassion may represent a broader regulatory process that interacts with mindfulness, interoceptive awareness, and emotion regulation systems. Individuals who exhibit a compassionate response to suffering might exhibit a greater willingness to confront painful emotions without resorting to avoidance or excessive engagement, thereby facilitating adaptive meaning-making following trauma (91). Such mechanisms may diminish the probability that transient post-traumatic distress evolves into chronic depressive symptomatology. It is crucial to note that self-compassion is increasingly recognized as a modifiable clinical objective. Interventions rooted in compassion-focused and mindfulness-based approaches have shown encouraging outcomes on emotional functioning, indicating that the enhancement of self-compassion may not only mitigate vulnerability but also actively foster resilient adaptation among individuals who have experienced trauma (92). However, whether improvements following compassion-based interventions are specifically attributable to self-compassion itself or to overlapping mechanisms such as mindfulness, acceptance, emotional awareness, and psychological flexibility remains an important empirical question.

### 7.3 Proposed conceptual framework

Based on the critically analyzed evidence, we propose a theoretical framework in which exposure to trauma instigates a sequence of interrelated cognitive and emotional responses that may ultimately lead to either depressive states or resilience. This framework was developed through an integrative thematic synthesis of converging findings across trauma research, cognitive vulnerability models, emotion regulation theories, and resilience literature. Rather than representing a confirmed causal model, it is intended as a hypothesis-generating framework that organizes potential pathways linking trauma exposure with divergent psychological outcomes. Within this framework, trauma exposure may initiate a cascade of cognitive and emotional adaptations and calls into question preexisting beliefs concerning the self, others, and the broader world. The subsequent trajectories of adjustment are posited to be significantly contingent upon the dynamic interaction between maladaptive and protective mechanisms. Specifically, exposure to trauma may heighten the propensity for engaging in recurrent negative thought patterns, particularly rumination, while concurrently undermining effective emotion regulation capacities. These two processes are theorized to interact in a bidirectional manner, although the temporal ordering of these processes remains to be established empirically. The resultant feedback loop may perpetuate negative affect, reinforce maladaptive self-assessments, and hinder the processing of trauma, thereby elevating the risk for depressive outcomes. In contrast, self-compassion is posited to serve as a protective moderator that operates at various stages of this pathway. Self-compassion may potentially buffer these associations by reducing self-criticism, shame-related processing, and rigid negative self-evaluations. Subsequently, it may disrupt the reciprocal cycle that connects rumination and emotional dysregulation by fostering emotional acceptance and cognitive flexibility. Ultimately, self-compassion may directly enhance resilience by promoting adaptive coping strategies, facilitating meaning-making, and restoring psychological equilibrium. This integrative framework reconceptualizes post-traumatic adaptation as a dynamic and multidimensional process, rather than merely a linear outcome of trauma exposure (**Figure 1**). This framework should be interpreted as a hypothesis-generating conceptual model derived from existing empirical evidence and theoretical perspectives, rather than as an empirically validated causal pathway. Future empirical investigations utilizing longitudinal and person-centered methodologies are requisite to examine the temporal sequencing and reciprocal influences among these constructs. Such inquiries may contribute to the formulation of personalized interventions designed to concurrently mitigate maladaptive processes and reinforce protective factors. Furthermore, self-compassion may not operate independently but may interact with related capacities, including mindfulness, interoceptive awareness, attachment-related security, and emotion regulation flexibility. Clarifying these overlapping pathways represents a critical direction for future research.

**Figure 1:**
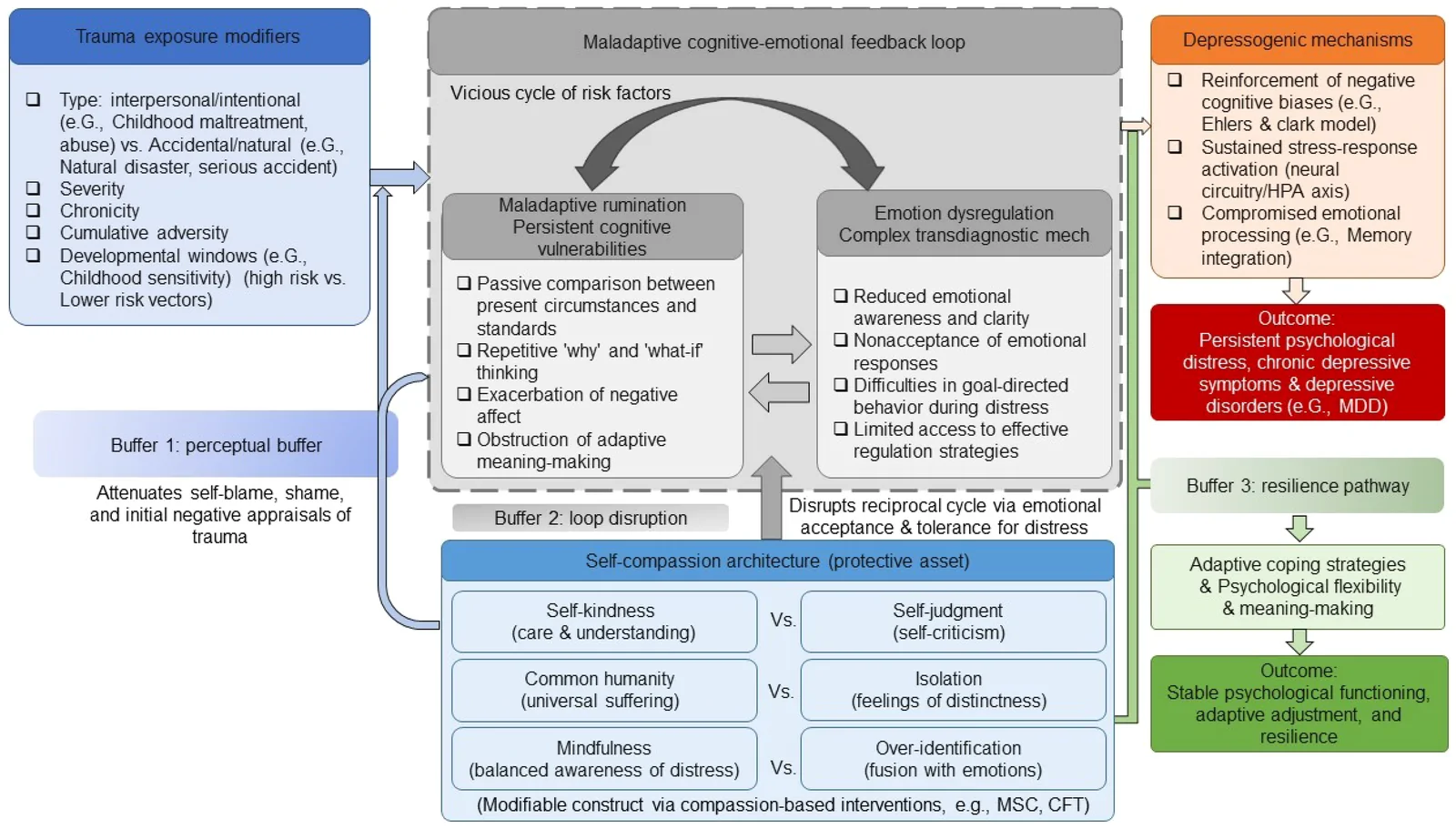
An integrative conceptual framework illustrating proposed pathways linking trauma exposure to psychological outcomes. The model highlights the reciprocal association between maladaptive rumination and emotion dysregulation as interacting cognitive-emotional vulnerability processes that may contribute to depressogenic trajectories following trauma exposure. Importantly, the framework depicts self-compassion as a potential protective resource through its multidimensional components (self-kindness, common humanity, and mindful awareness), which may attenuate maladaptive cognitive-emotional processes and facilitate adaptive psychological functioning. These pathways represent an integrative theoretical model derived from existing empirical evidence rather than established causal mechanisms. MDD, Major Depressive Disorder; MSC, Mindful Self-Compassion; CFT, Compassion-Focused Therapy.

## 8 Clinical implications

### 8.1 Screening and risk identification

The results analyzed in the current manuscript carry significant ramifications for the prompt identification of individuals who have experienced trauma and may possess an increased susceptibility to the onset of depressive disorders. Conventional evaluations conducted post-trauma predominantly emphasize the intensity of symptoms, with a particular focus on those associated with PTSD. Nonetheless, emerging research indicates that the assessment of fundamental cognitive and emotional mechanisms could markedly enhance the stratification of risk. Specifically, heightened levels of rumination, difficulties in emotion regulation, and lower self-compassion may represent clinically relevant vulnerability markers that are associated with increased risk for depressive symptoms following trauma exposure. Consequently, future trauma-informed assessment models may benefit from considering measures of repetitive negative thinking, emotion regulation difficulties, and self-relational processes alongside conventional symptom-based evaluations. Individuals who demonstrate ongoing rumination are especially predisposed to encounter enduring depressive symptoms and protracted recovery (93, 94) (**Table 4**). Trauma survivors exhibiting deficiencies in emotion regulation frequently manifest heightened psychological dysfunction and diminished functional outcomes (95, 96). In contrast, diminished levels of self-compassion have been persistently correlated with an increased susceptibility to trauma-related psychopathology and may serve to identify individuals who exhibit reduced capacity for adaptive self-soothing during distressing episodes (67). Notably, these variables may hold particular significance within high-risk populations, including survivors of childhood maltreatment, military veterans, emergency responders, and individuals subjected to recurrent interpersonal trauma. The implementation of comprehensive screening protocols that concurrently evaluate symptom severity and transdiagnostic vulnerability mechanisms may enhance the formulation of personalized intervention strategies and optimize the distribution of mental health resources. Recent developments in measurement-based care further indicate that ongoing assessment of cognitive-emotional processes throughout treatment may assist clinicians in the early identification of non-responsiveness and enable timely modifications to interventions as needed (97). Such approaches are consistent with emerging precision psychiatry models emphasizing individualized risk profiles rather than diagnosis alone (98) (**Figure 2**). However, the clinical utility and incremental predictive value of these transdiagnostic measures require further validation in longitudinal and diverse trauma-exposed populations.

**Table 4:** Clinical interventions targeting the proposed mechanisms.

Design/Sample	Intervention	Primary target mechanism	Main outcomes	Study
Open-label case series; *N* = 14 patients with residual depression	Rumination-Focused Cognitive Behavioral Therapy (RFCBT)	Rumination	Reduced depressive symptoms and rumination; 71% treatment response and 50% remission.	(93)
Phase II RCT; *N* = 121 adults with major depression	Concreteness Training (CNT) + treatment as usual	Abstract rumination, overgeneral thinking	CNT reduced rumination and depressive symptoms and improved long-term outcomes.	(94)
Randomized controlled trial; *N* = 58 women with childhood abuse-related PTSD	Skills Training in Affective and Interpersonal Regulation (STAIR) followed by exposure	Emotion dysregulation	Improved affect regulation, interpersonal functioning, and PTSD symptoms; gains maintained at follow-up.	(95)
RCT; *N* = 104 women with childhood abuse-related PTSD	STAIR + Exposure	Emotion dysregulation, interpersonal difficulties	Produced greater PTSD remission, enhanced emotion regulation, and lower dropout rates than comparison conditions.	(96)
Two-year RCT; *N* = 101 women with borderline personality disorder and suicidality	Dialectical Behavior Therapy (DBT)	Emotion dysregulation	Reduced suicidal behaviors, psychiatric hospitalization, and treatment dropout.	(101)
Pilot study and RCT; community adults (*N* = 21 and *N* = 52)	Mindful Self-Compassion (MSC) program	Self-compassion	Increased self-compassion, mindfulness, and psychological well-being; effects maintained up to 1 year.	(67)
Randomized intervention study; *N* = 126 smokers	Self-compassion training	Self-compassion, self-regulation	Accelerated behavioral change, particularly among highly self-critical individuals.	(103)
Multi-site randomized clinical trial protocol in veterans	Acceptance and Commitment Therapy (ACT)	Experiential avoidance, psychological flexibility	ACT proposed as a transdiagnostic intervention for trauma-related distress and comorbidity.	(104)
Longitudinal clinical study; veteran sample	Mindfulness-Based Stress Reduction (MBSR)	Mindfulness, emotion regulation, self-compassion	Improved PTSD symptoms, depression, acceptance, mindfulness, and quality of life.	(105)

ACT, Acceptance and Commitment Therapy; CNT, Concreteness Training; DBT, Dialectical Behavior Therapy; MBSR, Mindfulness-Based Stress Reduction; MSC, Mindful Self-Compassion; PTSD, posttraumatic stress disorder; RCT, randomized controlled trial; RFCBT, Rumination-Focused Cognitive Behavioral Therapy; STAIR, Skills Training in Affective and Interpersonal Regulation.

**Figure 2:**
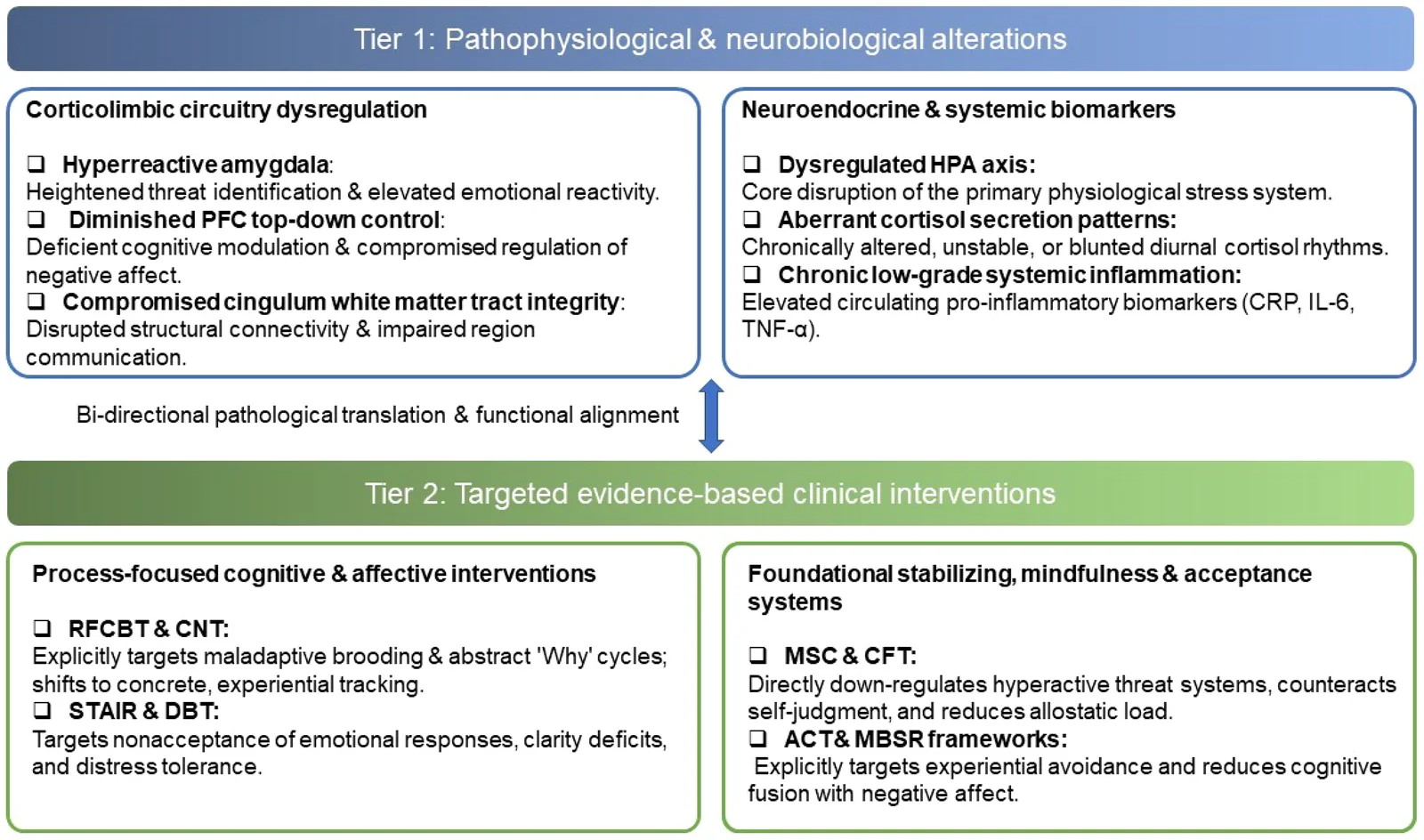
A translational conceptual matrix linking trauma-related psychological and neurobiological alterations (Tier 1) with potential evidence-informed clinical approaches (Tier 2). The bidirectional vectors represent hypothesized functional relationships between cognitive-emotional mechanisms, neural circuitry, stress-related biological systems, and therapeutic targets. This framework illustrates potential intervention pathways while acknowledging that direct mechanistic links between specific biomarkers and clinical outcomes require further empirical validation. ACT, Acceptance and Commitment Therapy; CNT, Concreteness Training; CFT, Compassion-Focused Therapy; CRP, C-Reactive Protein; DBT, Dialectical Behavior Therapy; HPA, Hypothalamic-Pituitary-Adrenal; IL-6, Interleukin-6; MBSR, Mindfulness-Based Stress Reduction; MSC, Mindful Self-Compassion; PFC, Prefrontal Cortex; RFCBT, Rumination-Focused Cognitive Behavioral Therapy; STAIR, Skills Training in Affective and Interpersonal Regulation; TNF-α, Tumor Necrosis Factor-alpha.

### 8.2 Targeting rumination in treatment

Given the substantial empirical evidence that correlates rumination with post-traumatic depressive disorders, therapeutic interventions that specifically target maladaptive repetitive cognitive processes may represent a promising approach. Traditional cognitive-behavioral methodologies often address detrimental cognitions; however, contemporary interventions increasingly prioritize the modification of the cognitive process rather than solely focusing on the content of thought. Rumination-Focused Cognitive Behavioral Therapy (RFCBT) was explicitly designed to mitigate maladaptive ruminative tendencies. In an initial case series comprising individuals experiencing residual depressive symptoms, RFCBT yielded considerable reductions in both depressive symptoms and rumination, with 71% of participants exhibiting clinically significant improvement and approximately half attaining remission (93). Instead of contesting the veracity of cognitive thoughts, RFCBT accentuates a transition from abstract, evaluative cognition to more concrete, experiential forms of cognitive processing (93). Similarly, concreteness training (CNT) has exhibited potential as a low-intensity intervention aimed at addressing depressogenic cognitive processes. In a randomized controlled trial, participants who received CNT alongside standard treatment exhibited considerable reductions in depressive symptoms, rumination, and overgeneralized thinking that were sustained at follow-up assessments (94). These findings suggest that modifying maladaptive cognitive styles may help reduce processes associated with persistent depressive symptoms after trauma. From a clinical standpoint, the incorporation of rumination-focused strategies into trauma treatment frameworks may prove particularly advantageous for individuals who find themselves ensnared in repetitive self-blaming or counterfactual cognitive patterns. Metacognitive interventions that specifically aim to address beliefs concerning the utility and uncontrollability of rumination have also revealed therapeutic promise within emotional disorders (99). Moreover, clinicians may advocate for behavioral activation and present-focused processing to mitigate excessive cognitive rumination and promote adaptive emotional involvement (100). Nevertheless, most evidence supporting rumination-focused interventions has been derived from depressive populations rather than specifically trauma-related depression. Therefore, their effectiveness among trauma survivors requires further investigation.

### 8.3 Improving emotion regulation skills

The current systematic review underscores the significance of emotion dysregulation as a pivotal transdiagnostic mechanism that interconnects trauma exposure with the development of depression and the hindrance of recovery processes. Consequently, the enhancement of emotion regulation capabilities may represent an important therapeutic target within trauma-focused therapeutic interventions. Empirical evidence derived from phase-based treatment modalities robustly corroborates this assertion. Cloitre et al. have demonstrated that the implementation of Skills Training in Affective and Interpersonal Regulation (STAIR), succeeded by exposure therapy, resulted in marked improvements in emotion regulation abilities, interpersonal functioning, and symptomatology of PTSD among individuals who have experienced childhood abuse (95). Subsequent randomized controlled trials have further elucidated that the combination of STAIR with exposure therapy leads to superior symptom alleviation, reduced attrition rates, and diminished adverse effects in comparison to treatments devoid of emotion regulation elements (96). Additional interventions that prioritize emotion regulation have also exhibited significant effectiveness. Dialectical Behavior Therapy (DBT), initially formulated for individuals with chronic suicidality, has consistently shown associations with decreases in self-injurious behaviors, psychiatric admissions, and emotional instability (101). While DBT was not specifically tailored for populations affected by trauma, its focus on distress tolerance, emotional awareness, and interpersonal efficacy makes it particularly pertinent for trauma survivors who experience profound affective dysregulation. However, evidence regarding DBT specifically for trauma-related depression remains less extensive than for other clinical populations. Consequently, clinicians may consider incorporating training in emotional identification, acceptance, distress tolerance, cognitive flexibility, and constructive emotional expression, particularly for individuals with marked emotion regulation difficulties. Furthermore, emerging research indicates that training in emotion regulation may augment responsiveness to subsequent trauma-focused exposure therapies by enhancing emotional preparedness and mitigating treatment attrition (102). These results bolster the rationale for sequential or phase-oriented treatment frameworks for individuals subjected to chronic or complex trauma.

### 8.4 Self-compassion-based interventions

Given that self-compassion has been associated with more adaptive psychological functioning following trauma, interventions targeting compassionate self-relating represent a promising but still developing area of clinical research. Among the most rigorously examined interventions is the Mindful Self-Compassion (MSC) program. In both pilot studies and randomized controlled trials, MSC has been shown to significantly enhance self-compassion, mindfulness, and psychological well-being, with these improvements sustained for up to one-year post-treatment (67). Importantly, because MSC integrates mindfulness practices with compassion training, improvements observed following MSC cannot always be attributed exclusively to self-compassion. Future studies using dismantling designs are needed to clarify the unique contribution of compassionate self-relating relative to mindfulness and other regulatory processes. These findings suggest that self-compassion-related capacities may be modifiable through structured interventions. It should be noted that improvements observed following compassion-based interventions cannot be assumed to reflect self-compassion alone, as these interventions often incorporate overlapping elements such as mindfulness, acceptance, and emotion regulation training. Therefore, future dismantling studies are required to determine whether compassionate self-relating provides unique therapeutic effects beyond these shared mechanisms. Compassion-Focused Therapy (CFT) similarly endeavors to alleviate feelings of shame and self-criticism while fortifying affiliative and soothing emotional systems. Preliminary findings suggest that integrating CFT components with cognitive-behavioral therapy may have potential benefits for individuals characterized by shame and self-criticism, although trauma-specific evidence remains limited (103). Self-compassion training may hold particular promise for individuals who have experienced trauma and who exhibit pronounced self-blame, guilt, or enduring shame. Third-wave cognitive-behavioral methodologies, encompassing Acceptance and Commitment Therapy (ACT) and mindfulness-oriented interventions, specifically address mechanisms that are critically pertinent to the recovery from trauma. ACT facilitates psychological flexibility and the acceptance of challenging internal experiences, which may lead to a diminishment of experiential avoidance and maladaptive cognitive fusion (91). Despite the limited availability of rigorous trauma-specific evidence, transdiagnostic ACT methodologies have demonstrated potential efficacy within veteran cohorts (104). Similarly, engagement in Mindfulness-Based Stress Reduction programs has been correlated with enhancements in PTSD symptomatology, depressive states, behavioral activation, and overall quality of life among veterans exposed to trauma (105). Collectively, these findings suggest that compassion-oriented and mindfulness-informed approaches may contribute to improvements in psychological functioning; however, the extent to which these effects are specifically mediated by self-compassion remains uncertain. Prospective treatment frameworks may gain from the incorporation of compassion-oriented strategies in conjunction with conventional trauma-focused therapies to concurrently reduce vulnerability factors and bolster protective capacities (92). A key issue for upcoming clinical research is pinpointing the active mechanisms that drive compassion-based interventions. While self-compassion is increasingly understood as a resilience-related capacity, its therapeutic benefits may stem from partially shared processes, such as mindfulness, interoceptive awareness, flexible emotion regulation, and decreases in self-critical thinking. Establishing these mechanisms will be crucial for designing targeted interventions and for identifying which trauma-exposed individuals are most likely to profit from compassion-focused approaches.

## 9 Future directions

Despite the accumulating evidence linking rumination, emotional dysregulation, and self-compassion to post-traumatic psychological outcomes, a number of significant gaps persist within the existing literature. However, the current evidence base remains limited by substantial methodological heterogeneity, the predominance of cross-sectional designs, and insufficient clarification of the mechanisms through which cognitive-emotional processes interact to shape divergent post-traumatic trajectories. Addressing these deficiencies may enhance the comprehension of the mechanisms that differentiate trauma survivors who develop depressive symptoms from those who exhibit resilience. First, there exists an urgent necessity for longitudinal investigations that explore the temporal dynamics among trauma exposure, cognitive-emotional processes, and psychological outcomes. Much of the current evidence is derived from cross-sectional methodologies, which restricts the ability to draw conclusions regarding causality and developmental pathways. Prospective research tracking individuals from the immediate aftermath of trauma through to long-term recuperation could clarify the temporal ordering and reciprocal influences among these mechanisms over time, rather than assuming a unidirectional pathway from trauma exposure to psychopathology. Second, subsequent inquiries should concentrate on clarifying the causal mechanisms that underpin the relationships among rumination, emotional dysregulation, self-compassion, and depression. Experimental and intervention-oriented studies may provide insights into whether altering these processes has a direct impact on resilience and depressive outcomes. Particular attention should be given to identifying the active mechanisms underlying self-compassion-based interventions. Future research should determine whether observed benefits are primarily attributable to compassionate self-relating itself or reflect overlapping processes shared with mindfulness, acceptance, interoceptive awareness, psychological flexibility, and broader emotion regulation capacities. Such mechanistically informed approaches are essential for distinguishing self-compassion as a specific regulatory process from related contemplative and cognitive-emotional constructs. Third, increased emphasis ought to be placed on cultural considerations. The majority of existing research has primarily focused on Western populations, thereby eliciting apprehensions regarding the extent to which findings can be generalized. Cultural conventions pertaining to emotional expression, self-construal, and coping mechanisms may significantly influence both susceptibility and resilience in the aftermath of trauma. Future studies should also examine whether the roles of rumination, emotion dysregulation, and self-compassion differ according to trauma type, developmental timing, gender, and socioeconomic context. Furthermore, developmental investigations are needed to clarify why some trauma-exposed individuals develop adaptive self-compassionate capacities whereas others experience persistent self-criticism, rumination, and emotional dysregulation. Factors such as attachment security, early caregiving experiences, trauma timing, interpersonal trauma exposure, and developmental disruption of emotional awareness may represent important moderators influencing these divergent trajectories. Moreover, future research endeavors would gain from the implementation of multilevel models that amalgamate psychological, social, and biological variables. Investigating the interactions among individual attributes, interpersonal dynamics, environmental stressors, and expansive sociocultural factors may yield a more holistic comprehension of post-traumatic adaptation. Furthermore, additional neurobiological inquiry is essential for delineating the neural and physiological correlates of rumination, emotional dysregulation, and self-compassion in populations exposed to trauma. Employing multimodal methodologies that integrate neuroimaging, psychophysiological assessments, and biomarkers may elucidate the biological pathways that connect trauma with depression and resilience. Importantly, future investigations should move beyond isolated brain regions and examine large-scale neural network interactions, including the DMN, SN, and interoceptive systems, which may provide a more integrated account of self-referential processing, emotional regulation, and resilience following trauma. The rapid proliferation of digital mental health interventions concurrently offers substantial opportunities for advancement. Mobile applications, internet-based therapeutic modalities, and ecological momentary assessment technologies may offer opportunities to enhance real-time monitoring and deliver scalable interventions targeting maladaptive rumination and emotion regulation difficulties, although their effectiveness and long-term clinical utility require further empirical validation. Moreover, innovations in personalized psychiatry may facilitate the development of more individualized methodologies for trauma recovery. The identification of unique cognitive-emotional profiles among survivors of trauma could yield insights that inform precision-oriented prevention and treatment strategies, potentially enabling interventions to be better tailored according to individual risk and protective profiles rather than diagnostic categories alone. Ultimately, future research should seek to empirically test the proposed integrative framework rather than assume a predetermined causal pathway. Person-centered longitudinal designs, experimental manipulation of cognitive-emotional processes, and mechanistically informed intervention studies will be essential to determine when rumination becomes maladaptive, when emotion regulation difficulties emerge as maintaining factors, and how protective capacities such as self-compassion interact with these vulnerabilities to promote resilience. Collectively, these avenues of research may enhance our understanding of post-traumatic depression and resilience in a more nuanced and mechanistic manner, ultimately guiding the creation of more effective and personalized therapeutic interventions.

## 10 Limitations of the current literature

Several constraints within the extant literature must be acknowledged when analyzing the interrelations among trauma exposure, rumination, emotion dysregulation, self-compassion, and post-traumatic outcomes. Primarily, the literature is marked by an overwhelming prevalence of cross-sectional studies, which significantly constrain inferences related to temporal sequencing and causal relationships. Although numerous investigations have documented meaningful correlations between maladaptive cognitive-emotional processes and depressive symptoms subsequent to trauma, cross-sectional methodologies do not allow for the ascertainment of whether rumination and emotion dysregulation precede depressive states, emerge as outcomes of such states, or mutually exacerbate each other over time. Likewise, the protective function of self-compassion cannot be unequivocally established in the absence of prospective evidence. Therefore, the proposed integrative framework should be interpreted as a theoretically informed model requiring empirical validation rather than as an established causal pathway. Second, there exists substantial methodological heterogeneity among the various studies. Discrepancies in trauma definitions, sample characteristics, assessment timing, and outcome measures render comparisons across investigations exceedingly complex. Investigations have explored a wide array of trauma populations, including individuals who have endured childhood maltreatment, interpersonal violence, combat exposure, natural disasters, and medical trauma, each of which may engage distinct psychological processes. Importantly, trauma characteristics such as developmental timing, chronicity, interpersonal nature, and severity may represent critical moderators that contribute to divergent psychological trajectories but remain inconsistently examined across studies. Furthermore, discrepancies in the operationalization of resilience and depression contribute to the variability in research findings. A further limitation concerns variability in methodological quality among available studies. Differences in study design, sample size, measurement approaches, and statistical control of relevant confounders introduce uncertainty regarding the strength and reliability of reported associations. Although many studies provide valuable insights, differences in methodological rigor should be considered when interpreting the cumulative evidence. An additional significant limitation is the pronounced dependence on self-report measures. The majority of studies evaluating rumination, emotion dysregulation, self-compassion, and depressive symptoms have predominantly utilized self-administered questionnaires. Such measures are potentially vulnerable to recall bias, social desirability influences, and shared method variance, which may lead to exaggerated associations among constructs. Future research endeavors would be enhanced by the integration of clinician-rated assessments, behavioral paradigms, ecological momentary assessment, and objective physiological measures. In particular, mechanistically oriented approaches are required to determine whether the beneficial effects attributed to self-compassion reflect compassionate self-relating specifically or overlapping processes associated with mindfulness, acceptance, interoceptive awareness, and broader emotion regulation abilities. Another important limitation is the incomplete characterization of the neurobiological mechanisms underlying these psychological processes. Although previous research has emphasized individual regions such as the amygdala and prefrontal cortex, emerging evidence suggests that trauma-related rumination and emotional dysregulation may involve interactions among large-scale neural networks, including the Default Mode Network, Salience Network, and interoceptive systems. Greater integration of network-based neuroscience approaches will therefore be necessary to clarify how cognitive, emotional, and bodily processes interact during trauma recovery. Finally, the extant corpus of evidence is hampered by insufficient cross-cultural representation. A significant proportion of investigations has been undertaken within Western, educated, industrialized, rich, and democratic populations, thereby prompting apprehensions regarding the generalizability of the findings to a variety of cultural contexts. Variations in cultural frameworks concerning emotional expression, coping mechanisms, self-related cognitions, and interpretations of traumatic experiences may significantly impact both vulnerability and resilience processes. Consequently, an enhanced inclusion of culturally diverse populations is imperative to cultivate a more nuanced and universally applicable comprehension of post-traumatic adaptation. Collectively, these limitations highlight the necessity for more methodologically rigorous, longitudinal, multimethod, and culturally attuned research to elucidate the mechanisms that underlie the divergent psychological outcomes that ensue following trauma.

## 11 Conclusion

Trauma exposure represents a major risk factor for depressive disorders; however, substantial heterogeneity exists in post-traumatic trajectories, with many individuals demonstrating resilience despite significant adversity. This narrative review addresses an important theoretical gap by integrating previously fragmented literatures on rumination, emotion dysregulation, and self-compassion within a unified cognitive-emotional framework of post-traumatic adaptation. Rather than conceptualizing trauma-related depression as a direct consequence of exposure alone, the proposed model highlights how maladaptive repetitive thinking and impaired emotional regulation may interact with protective self-relational capacities to shape vulnerability and resilience. Current evidence suggests that rumination and emotion dysregulation are consistently associated with greater depressive symptoms following trauma, whereas self-compassion represents a promising protective factor associated with adaptive coping and psychological adjustment. However, the available literature remains limited by methodological heterogeneity, predominance of cross-sectional designs, reliance on self-report measures, and uncertainty regarding causal relationships among these constructs. In addition, whether the beneficial effects attributed to self-compassion are uniquely related to compassionate self-relating or overlap with mindfulness and broader emotion regulation mechanisms remains insufficiently understood. Future research should empirically test this conceptual framework through longitudinal, experimental, and mechanism-focused studies capable of clarifying temporal relationships among trauma exposure, cognitive-emotional processes, and psychological outcomes. Person-centered approaches, culturally diverse samples, and intervention studies using dismantling designs may further determine for whom and under what conditions these mechanisms contribute to recovery or resilience. A more precise understanding of these pathways may ultimately facilitate the development of personalized, mechanism-informed interventions for individuals affected by trauma.
